# Chronic Lymphedema of the Lower Limb: A Rare Cause of Dislocation of Total Hip Arthroplasty

**DOI:** 10.7759/cureus.579

**Published:** 2016-04-20

**Authors:** Raju Vaishya, Amit Kumar Agarwal, Nishint Gupta, Vipul Vijay

**Affiliations:** 1 Orthopaedics, Indraprastha Apollo Hospitals; 2 Orthopaedics, St. Stephens Hospital

**Keywords:** total hip arthroplasty, dislocation, chronic lymphedema

## Abstract

Total hip arthroplasty (THA) in a patient with chronic lymphedema of both lower limbs is rarely reported in the literature. Chronic lymphedema is a challenging condition associated with various complications especially in a patient with THA. However, dislocation of the total hip prosthesis due to acute exacerbation of lower limb swelling in the postoperative period is an extremely rare complication. The cause that led to the dislocation of the prosthesis is intricate and difficult to assess, as this has not been discussed in the literature yet. We believe that the excessive weight of the limb due to chronic lymphedema had a deleterious effect on the biomechanics of total hip prosthesis, thereby increasing the tendency for dislocation. This case illustrates that chronic lymphedema of the lower limb should be dealt with aggressively using various modalities like intermittent pneumatic compression pumps and compression stockings after THA in such patients.

## Introduction

Acute dislocation of the total hip prosthesis is not an uncommon complication after total hip arthroplasty (THA) with an incidence of up to 6% reported in the literature [1]. These dislocations are usually treated by a closed reduction in acute settings, and an open reduction in other cases [2]. The common factors associated with dislocation of the hip prosthesis include malpositioning of the acetabular and femur components, surgical approach, the experience of the surgeon, and patient-associated factors like age, sex, neurological disorders, and previous surgery [3]. THA in a patient with chronic lymphedema is rare and hip dislocation associated with this condition has not yet been reported in the literature. We present a rare cause of dislocation of a hip prosthesis in a patient with chronic lymphedema and discuss the probable cause of the dislocation. Informed consent was obtained from the patient for this study.

## Case presentation

**Figure 1:**
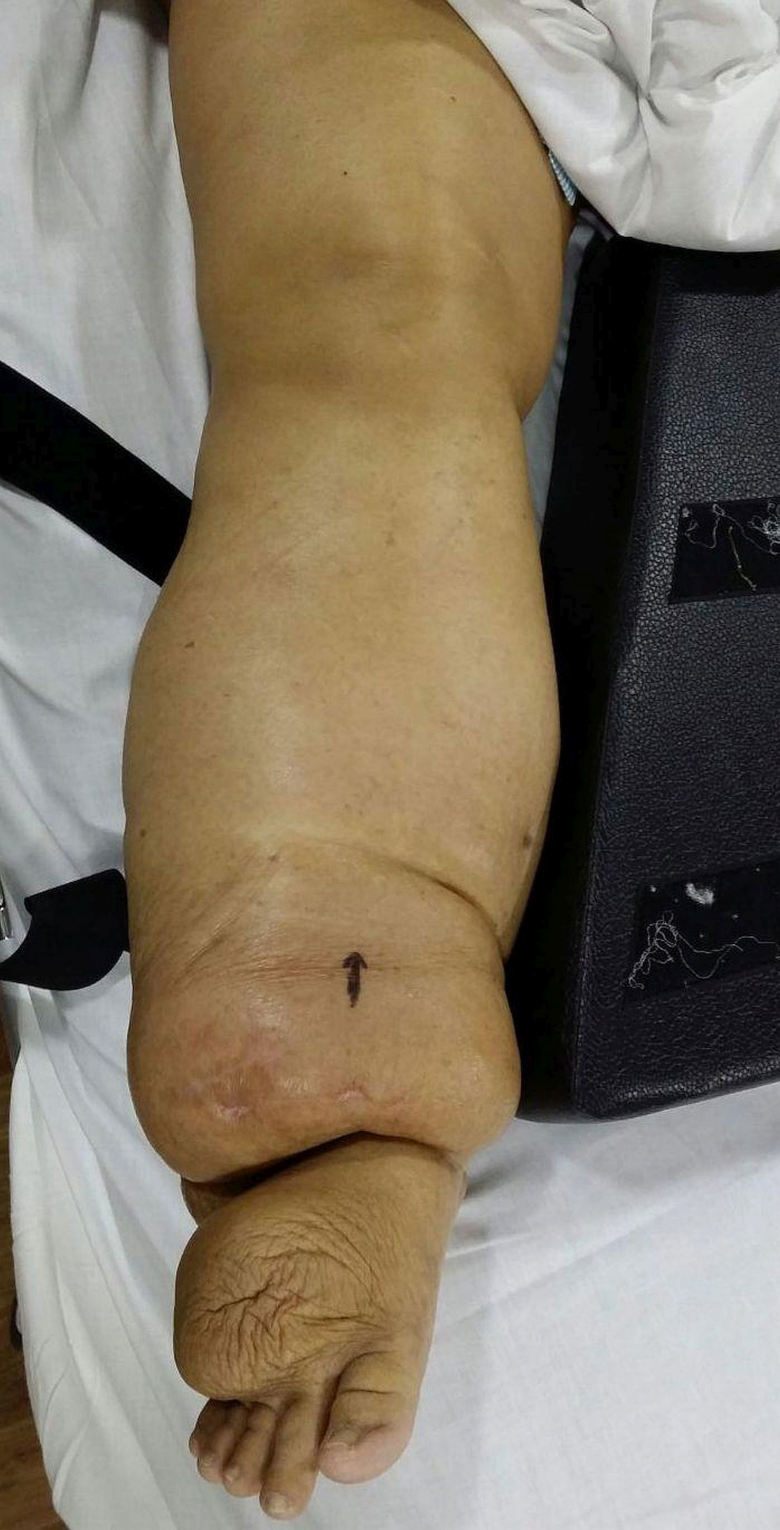
Clinical picture showing chronic lymphedema of the right lower limb.

A 52-year-old female presented to us with a limp and pain in the right hip joint that had been persisting for the last year. On general examination, she had associated chronic lymphedema of both the lower limbs due to an early filarial infection (Figure 1).

**Figure 2:**
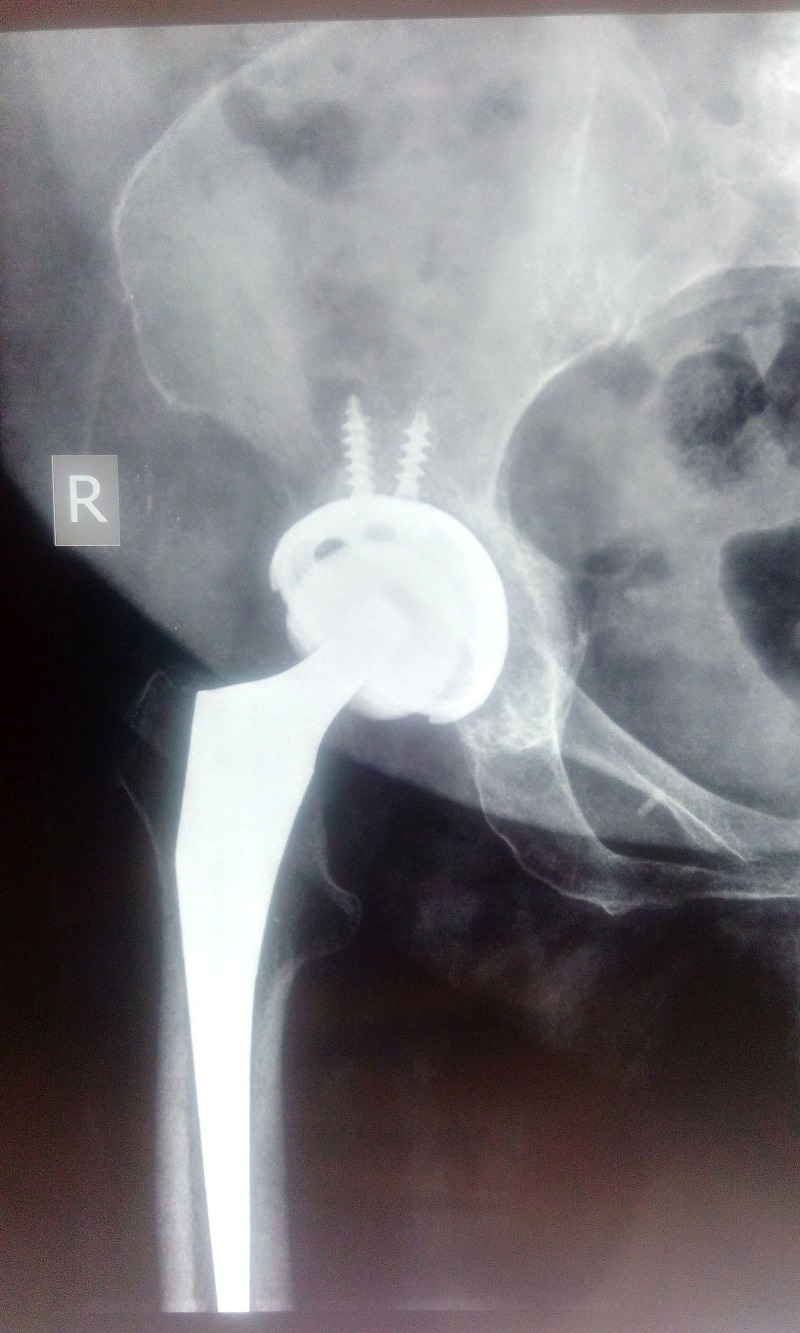
Immediate postoperative X-Ray of right hip. Anteroposterior view (AP) showing total hip replacement.

Plain radiographs revealed right hip osteoarthritic changes. A THA was done by modified Hardinge lateral approach [4] (Figure 2).

**Figure 3:**
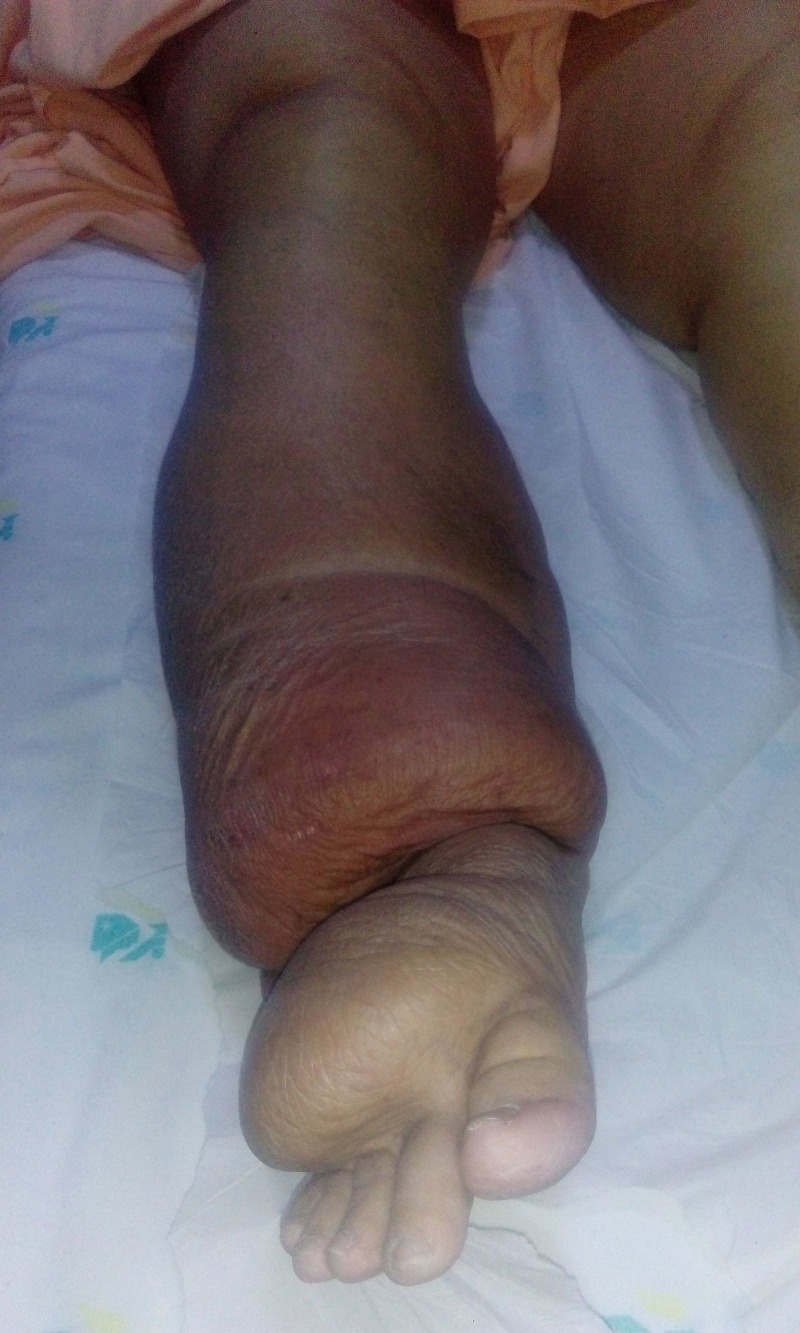
Note the shiny and tense skin associated with massive swelling of the whole right leg.

The stability of the prosthesis was checked intraoperatively by using a shuck test [5], and stability in flexion, abduction, adduction, and rotation was found to be good. In the postoperative period, the patient did early active ankle pump exercises and a passive range of motion exercises under supervision while maintaining proper positioning of the limb. Non-weight-bearing mobilization with a walking frame was permitted on the fifth postoperative day. The patient was discharged from the hospital after suture removal on the 11th postoperative day. Three weeks after the surgery, the patient reported that she experienced severe pain and deformity of the hip while getting out of bed for mobilization. On examination, we noted gross swelling of the lower limb due to acute exacerbation of chronic lymphedema (Figure 3), and that the right leg and ankle had increased to almost twice their preoperative size.

**Figure 4:**
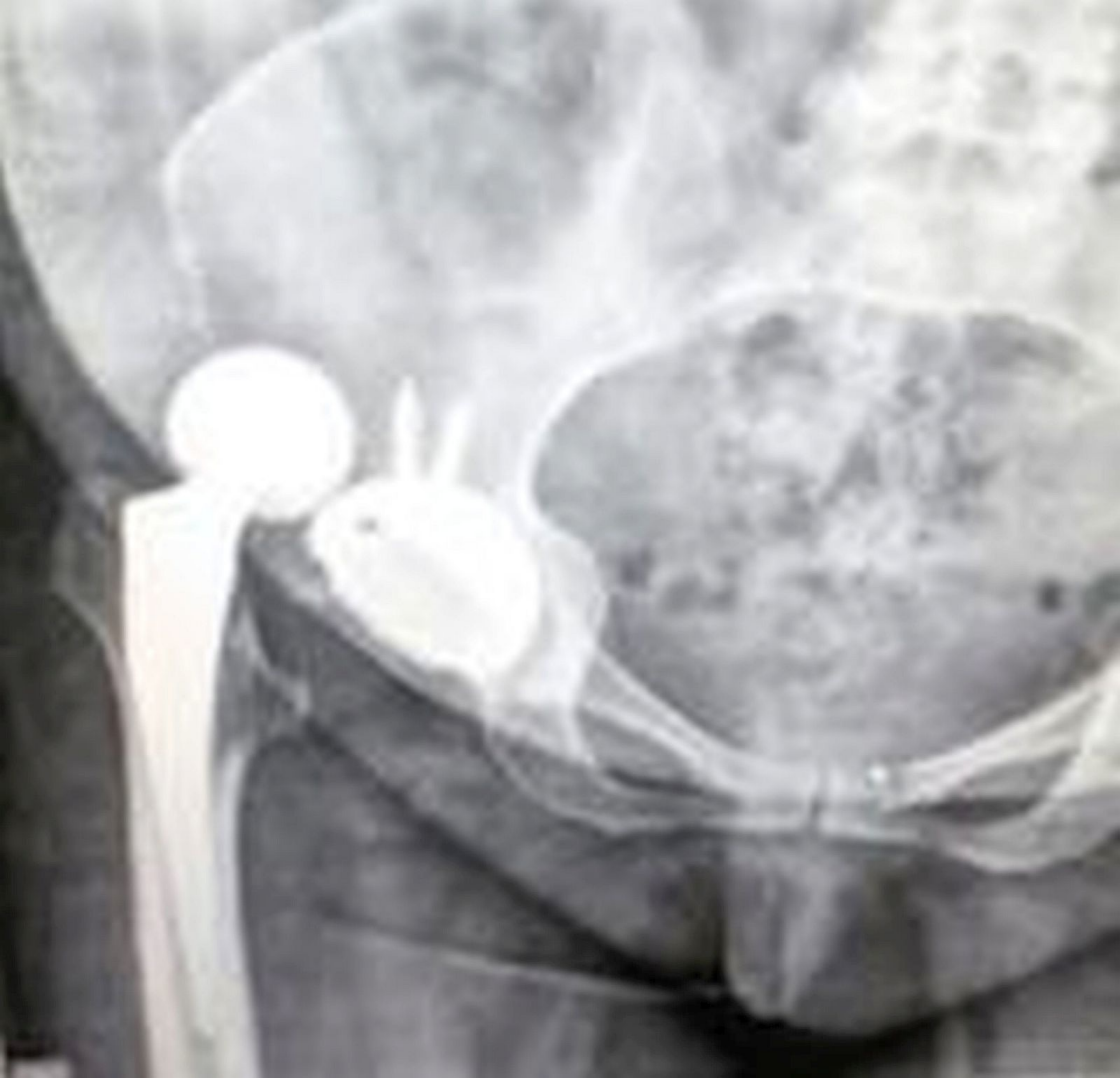
One month postoperative X-Ray of right hip. Anteroposterior view (AP) showing dislocated total hip replacement.

Radiographs showed posterior dislocation of the total hip prosthesis (Figure 4).

An attempt at manipulation by Allis’s method [6] under anesthesia achieved stable reduction of the dislocated prosthesis. The patient was given intermittent pneumatic compression pumping [7] and compression stockings to reduce the lower limb swelling [8]. The leg swelling responded well to this treatment and since then the hip has remained stable. The patient was walking without any problems at the one-year follow-up.

## Discussion

Chronic-lymphedema-associated problems of the lower limbs can be varied: superficial and deep ulcers, deep venous thrombosis, and increased time for rehabilitation [9]. Dislocation of the total hip prosthesis in chronic lymphedema patients may occur due to increased weight of the lower limb. However, the mechanism of dislocation in this particular situation has not been discussed in the literature. Relative immobolization and fluid retention in the postoperative period caused increased lymphedema and increased the weight of the operated lower limb. We believe that the excessive weight of the limb rendered the hip joint more prone to dislocation.

**Figure 5:**
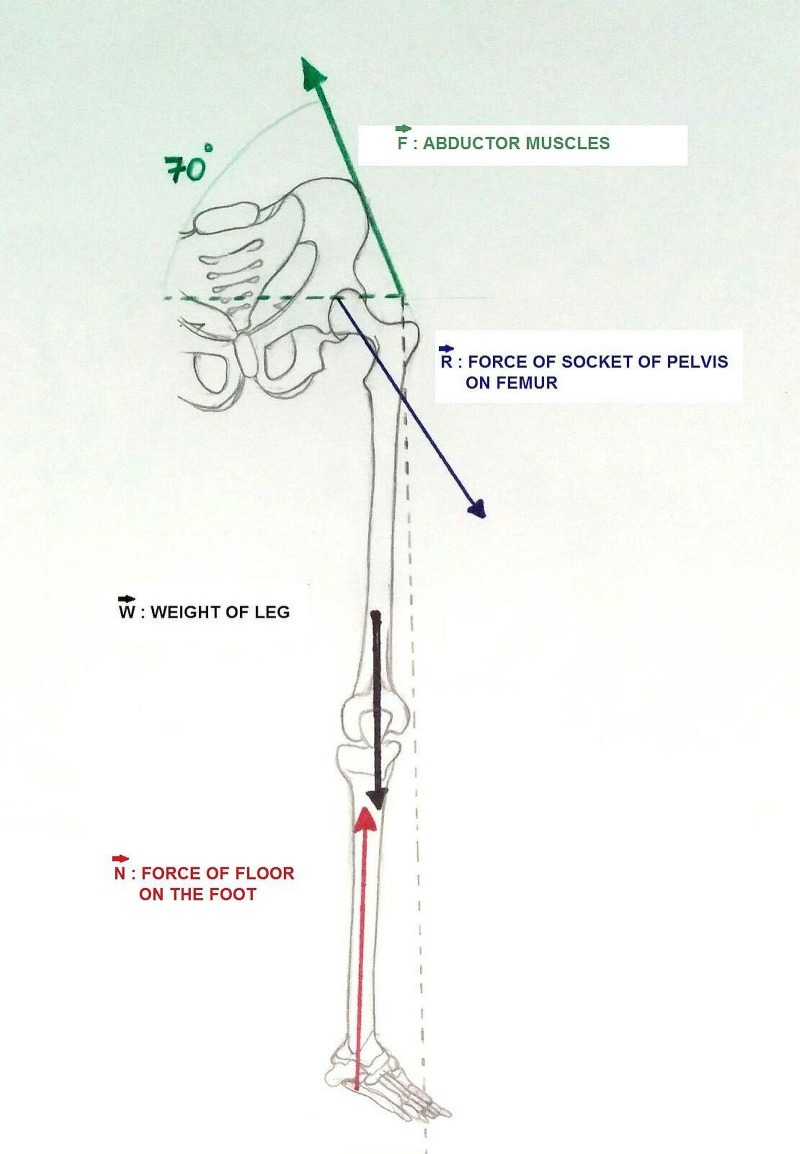
Line diagram showing the biomechanics around the hip.

Two unequal forces acting in opposite directions will bring about equilibrium only when the product of the magnitude of one force and its lever arm (the distance from its point of application from the fulcrum) is equal to the product of the magnitude of the other force and its lever arm [10]. It has been experimentally proved that the long lever arm requires smaller force to overcome the short lever arm. The increase in the weight of the limb, with the lever arm distance remaining constant, made it prone to dislocation at the hip when the patient was just trying to get out of bed. In our case, the excessive increase in the load (i.e. the weight of the leg), with the reduced strength of the abductors created an inequilibrium of the forces around the fulcrum, making it prone to dislocation (Figure 5).

## Conclusions

THA in a setting of chronic lymphedema of both lower limbs is a rare and unusual presentation. It is associated with a high risk of hip dislocation due to the altered biomechanics of the lower limb caused by increased postoperative lymphedema. Hence, there is a need for aggressive management of the postoperative swelling of the limb in chronic lymphedema patients to prevent the catastrophic complication of dislocation. This can be achieved by using an intermittent pneumatic compression pump, compression stockings, and starting early physiotherapy of the limb. Furthermore, adequate precautions must be taken during the mobilization of the patient.
